# A Parody

**Published:** 1906-10

**Authors:** Fritz Lanham

**Affiliations:** Austin, Texas


### A Parody.

BY FRITZ LANHAM, AUSTIN, TEXAS.

Once upon a midnight dreary I was dreaming, weak and weary,
In the yellow fever region, where I'd ofttimes dreamed before;
In my broken slumber napping, large mosquitoes I was slapping,
As my life-blood they were sapping while I nodded on the floor.
Twelve I struck,—quite out of order, for the clock lacked 12 a quarter,—
And a little crimson border round my budding face they wore;
"'Tis a serenade!" I stuttered, as between their stings I shuddered,
While they warbled as I muttered, "Only this and nothing more!"

Many hours I kept on blinking while they stimulated thinking
By their systematic soundings deep into my flaming nose;
Fagged and drowsy, I was musing on the germs they were infusing
As, a striking climax using, they aroused me from repose.
Eagerly I wished the morrow to secure surcease of sorrow

From the balms that I might borrow which the neighbors had in store;
But that crowd of suckers humming never ceased their constant drumming,
But with reinforcements coming kept it up forevermore.

As a sweet, inviting calling still they deemed my painful squalling,
And they came in force appalling as they had not come before;
Then I rose with fear and quaking, o'er my face my fingers raking
To dispel the awful aching they had made in search of gore.
Deep into the darkness peering, I discerned a light was nearing,
Confident the sun appearing cast the glow upon my floor;
But another swarm advancing through my window came a prancing,
Causing all that light entrancing,—lightning bugs and nothing more.

Back into the chamber turning, all my soul within me burning,
For the daylight I was yearning as I rattled and I swore;
But, despite my patient rubbing, still I felt that ruthless drubbing
Till I saw, as I kept scrubbing, beaming rays creep o'er the door.
Soon the day broke and was mended as the sun his course ascended,
And, my little vigil ended, I arose from off the floor;
Happy that the morning gleaming all about the chamber streaming
Gave surcease of my sad dreaming. Let me dream there nevermore.

---

Dr. D. L. Peeples, Navasota, major and surgeon Texas National Guard, has been elected professor of surgery in the College of Physicians and Surgeons, Dallas, Texas.

---

Dr. Z. T. Bundy, of Millford, Texas, has been appointed surgeon to the Confederate Soldiers' Home, Austin, Texas. Dr. Bundy was a Confederate soldier, serving with Forrest's Cavalry when a mere lad. This is a most excellent appointment.

---

"Last week a delinquent subscriber said he'd pay if he lived. He died.

"Another said he would see me tomorrow. He's blind.

"Still another said he'd pay me within a week or go to the devil. He went."

"There are hundreds," writes the country editor who read these lines, "who should take warning from these procrastinators and pay up now."—*Ex.*

